# Early onset sleep disorders predict severity, progression and death in multiple system atrophy

**DOI:** 10.1007/s00415-025-12969-6

**Published:** 2025-03-01

**Authors:** Giulia Giannini, Luca Baldelli, Federica Provini, Ilaria Cani, Simone Baiardi, Luisa Sambati, Franco Magliocchetti, Pietro Guaraldi, Piero Parchi, Pietro Cortelli, Giovanna Calandra-Buonaura

**Affiliations:** 1 IRCCS Istituto Delle Scienze Neurologiche Di Bologna, Ospedale Bellaria, Via Altura 3, 40139 Bologna, Italy; 2 Department of Biomedical and Neuromotor Sciences (DiBiNeM), Alma Mater Studiorum - University of Bologna, Bologna, Italy

**Keywords:** Multiple system atrophy, Cohort studies, Natural history studies (prognosis), Disease progression, Sleep disorders

## Abstract

**Background:**

Early stridor onset (≤ 3 years from disease onset) is a predictor of shorter survival in Multiple System Atrophy (MSA), but its role on disease progression is not yet established. In MSA, previous studies on trajectories of disease did not include stridor and REM sleep behavior disorder (RBD) as clinical variable.

The aims of the study were: (1) to investigate disease progression in MSA patients with early stridor onset and with early stridor and/or RBD onset; (2) to assess cerebrospinal fluid (CSF) levels of neurofilament light chain protein (NfL) in MSA patients with early onset sleep disorders.

**Methods:**

This is a retrospective and prospective cohort study including 208 (120 males) MSA patients. Occurrence of symptoms/signs, milestones of disease progression, and their latency from disease onset were collected. RBD and stridor were video-polysomnography (VPSG)-confirmed. CSF NfL levels were analyzed. Survival data and predictors of mortality were calculated.

**Results:**

Out of 208 MSA patients (157 deceased), 91 were diagnosed with stridor and 160 with VPSG-confirmed RBD. Patients with early stridor onset (*n* = 41) and with early stridor and/or RBD onset (*n* = 132) showed an early autonomic involvement, developed a more progressive and severe disease and presented higher CSF NfL than those with late stridor and RBD onset. Early stridor and early RBD were independent risk factors on MSA survival.

**Conclusions:**

The evidence of a more rapid and severe disease progression and of high CSF NfL levels in patients who early developed sleep disorders could define a different MSA phenotype with a widespread impairment of central-brainstem circuits.

**Supplementary Information:**

The online version contains supplementary material available at 10.1007/s00415-025-12969-6.

## Introduction

Multiple system atrophy (MSA) is a rare, sporadic, neurodegenerative disorder characterized by a combination of autonomic failure, cerebellar syndrome and poorly levodopa responsive parkinsonism^1^. The most recent diagnostic criteria define 3 degrees of certainty for diagnosis (clinically probable, clinically established, and neuropathologically established) and 2 phenotypes: parkinsonian (MSA-P) or cerebellar (MSA-C), according to the predominant feature at the time of evaluation [1].

MSA is a severe disease, usually rapidly progressive, with a mean survival ranging from 6.2 to 10 years from onset [2–5]. However, long-survival forms have also been described [6, 7].

The heterogeneity of disease presentation and progression, along with the relatively short disease course from symptom onset to death, complicates targeted treatment of MSA. Improving the understanding of disease progression and factors affecting the disease course in MSA could be useful, in view of future disease-modifying therapies, to select more homogeneous subgroups of patients for clinical trials.

Studies on disease trajectories and on variables affecting survival focused on gender, age, MSA subtype, autonomic onset, and autonomic and/or motor impairment measured by specific scales [2–5, 8]. Despite a high frequency of disorders during sleep is reported in MSA, like REM Sleep Behavior Disorder (RBD) and sleep-related stridor [9–11], disease progression and trajectories in patients with early sleep disorders are scanty.

We previously demonstrated that MSA patients with RBD predating disease onset showed a more rapid disease progression [12]. Further, MSA patients presenting sleep-related stridor within 3 years of disease onset showed a worse prognosis compared to patients developing this symptom later, identifying early stridor as an independent risk factor for shorter survival [5, 10].

In the present study, we aimed to determine whether early onset stridor in MSA is also positively correlated with disease severity and more rapid disease progression, or shorter survival is simply a consequence of sudden event like sudden death. Further, we investigate disease severity and progression in MSA patients with early sleep disorder onset combining data on patients with early stridor onset and/or early RBD onset (predating and within 3 years from disease onset).

Finally, we aimed to assess if MSA patients with early onset of sleep disorders showed a higher level of cerebrospinal fluid (CSF) neurofilament light chain (NfL), a reliable biomarker of disease severity in MSA [13].

## Methods

### Study population and methods

This is a retrospective and prospective cohort study including 208 patients, enrolled at the Movement and Autonomic Disorders Clinic of the University of Bologna (MSA-BO cohort) [5], with a diagnosis of MSA, according to international criteria [1]. In the prospective cohort, 82 patients were recruited from two prospective studies and evaluated every 6 months from enrollment during the follow-up (BoProPark Study, RFPS2006-7–336,374, CE:09070; Natural History of Multiple System Atrophy, CE:17,093) [5]. In the retrospective cohort, we included 126 patients, referred to our Department between 1991 and October 2017, with a clinical diagnosis of MSA, evaluated at least once a year during the disease course. Three neurologists expert in movement disorders (PC, GC-B, PG) independently confirmed the diagnosis of MSA from data available at the last follow-up evaluation. Data were collected as previously described [5].

Patients were categorized as neuropathologically established, clinically established or clinically probable MSA, and classified as MSA-P or MSA-C based on the predominant motor involvement at the time of the last follow-up visit [1].

Occurrence of symptoms and signs [parkinsonism, cerebellar and pyramidal involvement, orthostatic hypotension (OH), urinary symptoms, sleep-related stridor, and RBD] and latency from disease onset were recorded from clinical history and neurologic examination. Symptoms and signs were categorized as early if predating disease onset or presenting within 3 years of disease onset.

Timing and latency of the following milestones of disease progression were recorded: frequent falls, wheelchair dependence, severe dysphagia or percutaneous endoscopic gastrostomy, severe dysarthria, and urinary catheterization [14]. Frequent falls were defined when at least 3 falls per year or frequent/several falls per year, related to motor impairment (parkinsonian or cerebellar), were documented.

The following instrumental/laboratory tests were analyzed, when available: (1) brain MRI or CT (if MRI was not possible); (2) neuropsychological evaluation [15, 16]; (3) video-polysomnography (VPSG) [17]; (4) head-up tilt test and other cardiovascular reflex tests [18]; (5) effect of levodopa (if applicable) assessed by a) a standardized oral levodopa kinetic-dynamic test, b) improvement of part III of the Unified Parkinson’s Disease Rating Scale after increasing levodopa up to 1 g/die [19]; (6) cardiac 123I-metaiodobenzylguanidin (MIBG)-SPECT; (7) cerebral 123I-ioflupane-SPECT; (8) CSF NfL levels, measured by a validated commercial enzyme-linked immunosorbent assay (ELISA) (NfL ELISA kit, IBL, Hamburg, Germany) [20].

RBD [21] and sleep-related stridor [10] were VPSG-confirmed. RBD onset was defined as the onset of the symptoms suggestive of RBD reported by the bed partner. In case of event registration during VPSG, the video was shown to the bed partner to ensure that the behavior was the same reported in medical history.

Time of stridor treatment (CPAP or tracheostomy) and latency of occurrence from stridor onset were collected. For patients treated with CPAP, tolerability of device and compliance (≥ 4 h per night) were collected.

Survival data were defined on the basis of time to death from the first symptom of disease. Causes of death were collected from medical reports, when available. Disease duration was defined as the interval in years from first symptom onset to death or to the end of this study.

Patients and/or their relatives were contacted by telephone and questioned regarding the clinical course and the time and the cause of death (if applicable) when the patient missed a clinical evaluation within 12 months.

### Statistical analysis

All clinical and instrumental data were collected using an ad hoc anonymized and standardized form and entered into an ad hoc database for statistical analysis.

Normality of continuous parameters distribution was checked using the Skewness-Kurtosis test. Variables were expressed as mean ± standard deviation (SD) or median and interquartile range (IQR), when appropriate. The *t* test or the Wilcoxon rank-sum test was performed to compare continuous variables as appropriate. Categorical variables were described by their absolute and/or relative frequencies and compared using chi-square test.

We performed Kaplan–Meier curves to graphically analyze the overall death survival from disease onset, and the log-rank test to compare survival between patient subgroups.

Survival data were defined based on time to death from the first symptom of disease. Univariate Cox regression analysis was performed studying the following variables: age at disease onset, sex, predominant clinical phenotype, first domain of onset (autonomic, parkinsonian, cerebellar), early stridor onset, early RBD onset, early autonomic onset (symptomatic OH, urinary urgency/frequency, urinary retention, and urinary incontinence), and CSF NfL levels.

Parameters with a value of *p* < 0.1 on univariate analysis were entered into the multivariable model.

A p value lower than 0.05 (2-sided) was considered significant. Statistical analysis was performed using the statistical software STATA^®^, version 17.0.

### Standard protocol approvals, registrations, and patient consents

The study was conducted in agreement with the principles of good clinical practice. The study protocol was approved by the local ethics committee of the local health service of Bologna, Italy (Cod. CE: 09070 and 17093). All patients gave written informed consent for study participation.

## Results

A total of 208 patients with MSA (120 males, 98 MSA-C and 110 MSA-P) were included in the study (6 neuropathologically established, 174 clinically established, and 28 clinically probable). Mean age at disease onset was 57.6 ± 8.5 years, mean disease duration was 7.5 ± 3.8 years. At the time of the analysis, 157 (75.5%) were deceased. The most common causes of death were sudden death (death during sleep, respiratory failure and cardio-respiratory arrest), bronchopneumonia and urinary infection. Demographic and clinical features of the study sample are shown in Table 1.

**Table 1:** Demographic and clinical characteristics of the study sample

		MSA MOTOR SUBTYPE		
	Total MSA sample	MSA-C	MSA-P	*p* value
	208			
		98	110	
Sex				
Male, *n (%)*	120 (57.7)	61 (62.2)	59 (53.6)	0.210
Female, *n (%)*	88 (42.3)	37 (37.8)	51 (46.4)	
Age at onset, *y*	57.6 ± 8.5	56.9 ± 7.6	58.3 ± 9.2	0.2430
Disease duration, *y*	7.5 ± 3.8	7.4 ± 3.9	7.6 ± 3.7	0.6857
Died				
Yes, *n (%)*	157 (75.5)	72 (73.5)	85 (77.3)	0.524
No, *n (%)*	51 (24.5)	26 (26.5)	25 (22.7)	
Long survival^1^, *n (%)*	11 (5.3)	5 (5.1)	6 (5.5)	0.910
Symptom of disease onset				
Autonomic, *n (%)*	115 (55.3)	56 (57.1)	59 (53.6)	0.953
Cerebellar, *n (%)*	70 (33.7)	65 (66.3)	5 (4.6)	** < 0.001**
Parkinsonism, *n (%)*	71 (34.1)	7 (7.1)	61 (58.2)	** < 0.001**
Milestones of disease progression				
Frequent^2^ falls, *n (%)*	117 (56.3)	54 (55.1)	63 (57.3)	0.336
Wheelchair dependence, *n (%)*	118 (56.7)	68 (64.3)	55 (50.0)	0.121
Urinary catheterization, *n (%)*	86 (41.4)	42 (42.9)	44 (40.0)	0.924
Unintelligible speech, *n (%)*	61 (29.3)	31 (31.6)	30 (27.3)	0.838
Severe dysphagia/PEG, *n (%)*	49 (23.6)	22 (22.5)	27 (24.6)	0.536
Stridor, *n (%)*	91 (47.4)	46 (48.9)	45 (45.9)	0.675
Stridor at onset, *n (%)*	10 (4.8)	8 (8.2)	2 (1.8)	**0.033**
Latency of stridor onset, *y*	4 (2–6)	3 (1–5)	4 (3–6)	**0.0154**
Disease duration after stridor onset, *y*	3 (1–5)	4 (2–6.5)	2 (1–4)	**0.0174**
History of RBD, *n (%)*	166 (79.8)	82 (83.7)	84 (76.4)	0.445
VPSG-confirmed RBD^3^, *n (%)*	160 (76.9)	78 (79.6)	82 (74.6)	0.064
Latency of RBD onset, *y*	0 [(− 2) – 2]	0 [(− 2) – 1]	0.5 [(− 2) – 3]	0.1186
CSF NfL^4^, *pg/ml*	3009 (2063–4216)	3127 (2033–4448)	2697.5 (2075.5–4200.5)	0.6026
Latency of CSF NfL^4^, *n*	4 (3–6)	3 (2–5)	4.5 (3–7)	0.0565

Data are expressed as mean ± standard deviation or median (interquartile range)

Statistically significant *p* values are denoted in bold

^1^ = disease duration ≥ 15 years

^2^ = frequent was defined at least 3 falls per year or documentation of frequent or several falls

^3^ = 180 patients underwent VPSG

^4^ = cerebrospinal fluid sample was collected in 87 patients

*CSF* cerebrospinal fluid, *MSA* Multiple System Atrophy, *MSA-C* multiple system atrophy with predominant cerebellar phenotype, *MSA-P* multiple system atrophy with predominant parkinsonism phenotype, *n* sample size, *NfL* neurofilament light chain, *PEG* percutaneous endoscopic gastrostomy, *RBD* REM sleep behavior disorder, *VPSG* video-polysomnography, *y* years

On the total sample, 180 patients underwent VPSG for stridor and/or RBD suspicion at history taking.

Overall, 91 (47.4%) patients were diagnosed with stridor during sleep, 41 patients were identified with early stridor onset, 10 of these presented stridor as first symptom of disease. One patient showed both stridor during sleep and wakefulness. Median latency of stridor onset was 4 (2–6) years and median disease duration after stridor onset was 3 (1–5) years. Concerning stridor treatment, 28 patients were treated with tracheostomy and 36 with CPAP, while 27 patients did not receive treatment. Among patients without treatment 4 patients did not tolerate CPAP and refused tracheostomy, 11 patients refused any treatment or otorhinolaryngology visit, 1 patient died 5 days after stridor diagnosis (before CPAP titration), while 11 for unknown reasons (deceased from 1992 to 1997, missing data).

RBD diagnosis was confirmed in 160 patients (76.9% of the total sample), 127 of these were identified with early RDB.

The median of CSF NfL levels, available in 87 MSA patients, resulted of 3009 (2063–4216) *pg/ml*. CSF collection for NfL was performed, for each patient, during the first inpatient evaluation, with a median latency from disease onset of 4 (3–6) years.

### Comparison between MSA patients with early and late stridor onset

Features of the MSA population with early and late stridor onset are compared in Table 2.

**Table 2:** Clinical features, latency of signs/symptoms onset and milestones of disease progression in MSA patients with stridor

		MSA patients with Stridor		
	Total sample with stridor	Sample with early stridor onset (≤ 3 years)	Sample with late stridor onset (> 3 years)	*p* value
	91	41	50	
Males,* n (%)*	48 (52.8)	24 (58.5)	24 (48.0)	0.316
Age at MSA onset, *y*	56.7 ± 8.8	59.3 ± 8.5	54.6 ± 8.5	**0.0114**
Died,* n (%)*	68 (74.7)	32 (78.1)	36 (72.0)	0.509
Disease duration, *y*	7.6 ± 3.8	5.8 ± 2.4	9.1 ± 4.2	**0.0001**
Long survival ^1^, *n (%)*	6 (6.6)	0 (0.0)	6 (12.0)	**0.022**
MSA subtype				
MSA-P,* n (%)*	45 (49.4)	17 (41.5)	28 (56.0)	0.168
MSA-C, *n (%)*	46 (50.6)	24 (58.5)	22 (44.0)	
Symptoms at MSA onset				
Parkinsonism, *n (%)*	22 (24.2)	8 (19.5)	14 (28.6)	0.319
Cerebellar, *n (%)*	25 (27.5)	11 (26.8)	14 (28.0)	0. 854
Autonomic, *n (%)*	60 (65.9)	32 (78.1)	28 (56.0)	**0.036**
Stridor treatment				
Treated with tracheostomy, *n (%)*	28 (30.8)	15 (36.6)	13 (26.0)	0.352
Treated with CPAP, *n (%)*	36 (39.5)	13 (31.7)	23 (46.0)	
No treated, *n (%)*	27 (29.7)	13 (31.7)	14 (28.0)	
Latency for stridor treatment, *n (%)*	0 (0–1)	0.5 (0–2)	0 (0–1)	0.1824
Symptoms during the disease course				
Parkinsonism, *n (%)*	82 (90.1)	35 (85.4)	47 (94.0)	0.170
Latency of parkinsonism, *y*	3 (0–4)	2 (1–3)	3 (0–5)	0.3025
Cerebellar, *n (%)*	78 (85.7)	35 (85.4)	43 (86.0)	0.547
Latency of cerebellar symptoms, *y*	2 (0–4)	2 (0–3)	2.5 (0–4)	0.3619
Pyramidal signs, *n (%)*	73 (80.2)	33 (80.5)	40 (80.0)	0.890
Latency of pyramidal signs, *y*	4 (3–6)	3 (3–4)	5 (3–6)	**0.0019**
Urinary urgency/frequency, *n (%)*	80 (87.9)	35 (85.4)	45 (90.0)	0.331
Early urgency/frequency onset, *n (%)*	68 (74.7)	35 (85.4)	33 (66.0)	**0.034**
Latency of urinary urgency/frequency, *y*	1 (0–2)	0 (0–1)	2 (0–4)	**0.0050**
Urinary retention, *n (%)*	57 (62.6)	24 (58.5)	33 (66.0)	0.482
Early urinary retention onset, *n (%)*	39 (42.9)	20 (48.8)	19 (38.0)	**0.039**
Latency of urinary retention, *y*	1.5 (0–4)	1 (0–2.5)	3 (0–5)	0.0712
Urinary incontinence, *n (%)*	61 (67.0)	25 (61.0)	36 (72.0)	0.338
Early urinary incontinence, *n (%)*	30 (32.9)	15 (36.6)	15 (30.0)	0.159
Latency of urinary incontinence, *y*	3 (1–6)	2 (0–4)	4 (2–8)	**0.0047**
Symptomatic OH, *n (%)*	72 (79.1)	36 (87.8)	36 (72.0)	**0.048**
Early Symptomatic OH onset, *n (%)*	49 (53.8)	28 (68.3)	21 (42.0)	**0.012**
Latency of symptomatic OH, *y*	3 (1–4)	2 (0.5–3)	3 (0–5)	0.1846
History of RBD, *n (%)*	79 (86.8)	35 (85.4)	44 (88.0)	0.348
VPSG-confirmed RBD, *n (%)*	84 (92.3)	37 (90.2)	47 (94.0)	0.117
Early VPSG-confirmed RBD onset, *n (%)*	71 (78.0)	36 (87.8)	35 (70.0)	**0.004**
Latency of RBD, *y*	0 [ (− 3)–1]	− 1 [ (− 3) − 1]	0 [ (− 2.5)− 2]	0.0852
Milestone of disease progression				
Frequent^2^ falls, *n (%)*	57 (62.6)	21 (51.2)	36 (72.0)	0.056
Latency of frequent falls, *y*	4 (2–6)	3 (2–4.5)	4 (3–6)	**0.0390**
Urinary catheterization, *n (%)*	45 (49.5)	17 (41.5)	28 (56.0)	0.251
Latency of urinary catheterization, *y*	5 (3–6)	3 (1–4)	5.5 (4–8)	**0.0004**
Unintelligible speech, *n (%)*	32 (35.2)	9 (22.0)	23 (46.0)	**0.026**
Latency of unintelligible speech, *y*	6 (5–8)	5 (4–5)	7 (5–8)	**0.0122**
Dysphagia/PEG, *n (%)*	30 (33.0)	6 (14.6)	24 (48.0)	**0.001**
Latency of dysphagia/PEG, *y*	7 (5–10)	4 (4–5)	8 (5–10)	**0.0289**
Wheelchair dependency, *n (%)*	61 (67.0)	24 (58.5)	37 (74.0)	0.212
Latency of wheelchair dependency, *y*	6 (4–8)	4 (3–5)	7 (5–8)	**0.0003**
CSF NfL^3^*, pg/ml*	3334 (2420–4604)	3825 (2420–4903)	3169.5 (2399–4448)	0.0980
Latency of CSF NfL^3^, *n*	4 (3–6)	3 (3–4)	5 (3–6)	0.2135

Data are expressed as n (%), mean ± SD, or median (interquartile range)

Statistically significant *p* values are denoted in bold (*p* value ≤ 0.05)

^1^ = disease duration ≥ 15 years

^2^ = frequent was defined at least 3 falls per year or documentation of frequent or several falls

^3^ = cerebrospinal fluid sample was collected in 45 patients

*CSF* cerebrospinal fluid, *MSA* multiple system atrophy, *MSA-C* multiple system atrophy with predominant cerebellar phenotype, *MSA-P* multiple system atrophy with predominant parkinsonism phenotype, *n* sample size, *NfL* neurofilament light chain, *OH* orthostatic hypotension, *PEG* percutaneous endoscopic gastrostomy, *RBD* REM sleep behavior disorder, *VPSG* video-polysomnography, *y* years

Compared to patients with late stridor onset, patients with early stridor onset showed a higher age at disease onset (59.3 ± 8.5 vs. 54.6 ± 8.5, *p* = 0.0114) and more frequently presented with autonomic onset (78.1% vs. 56.0%, *p* = 0.036). Disease duration was shorter in patients with early stridor onset (5.8 ± 2.4 vs. 9.1 ± 4.2, *p* = 0.0001).

No difference between the two groups was found concerning stridor treatment and latency for stridor treatment.

During the disease course, occurrence of symptomatic OH was more frequently reported in early stridor onset group than late stridor onset one (87.8% vs. 72.0%, *p* = 0. 048).

**Fig. 1:**
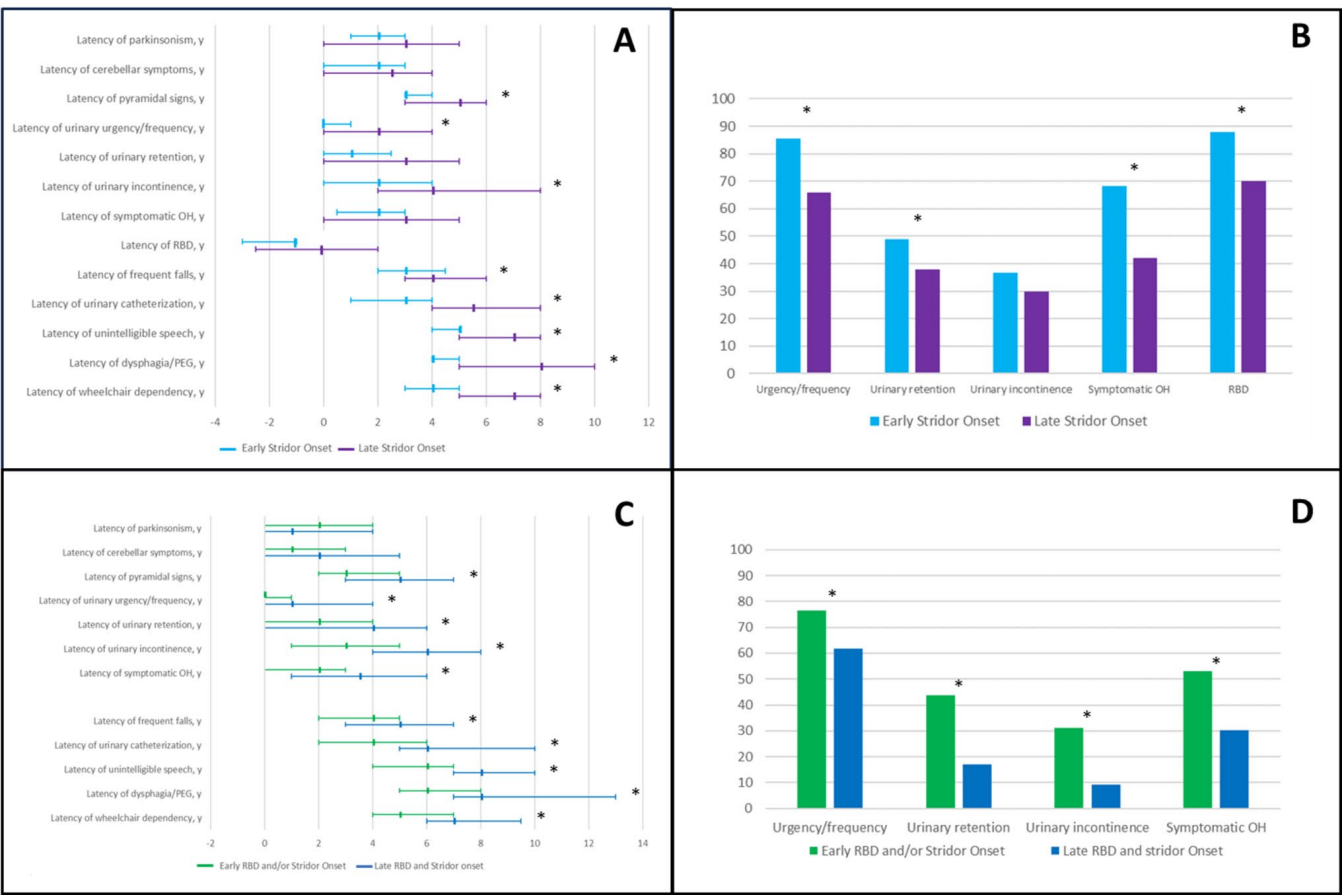
Disease progression in Multiple System Atrophy patient subgroups. **A** Latencies of symptoms/signs onset and milestones of disease progression in patients with early and late stridor onset; **B** early (presenting within 3 years of disease onset) symptoms and signs in patients with early and late stridor onset; **C** Latencies of symptoms/signs onset and milestones of disease progression in patients with early onset of stridor and/or RBD and in patients with late onset of stridor and RBD; **D** early (presenting within 3 years of disease onset) symptoms and signs in patients with early onset of stridor and/or RBD and in patients with late onset of stridor and RBD. *Data are expressed as median and interquartile range in years.* * = *p* value < 0.05 (statistically significant); *OH* orthostatic hypotension, *PEG* percutaneous endoscopic gastrostomy, *RBD* REM sleep behavior disorder, *y* = years

Patients with early stridor onset, when compared to those with late stridor onset, showed an earlier onset of pyramidal signs [3 (3–4) vs. 5 (3–6), *p* = 0.0019], urinary urgency/frequency [0 (0–1) vs. 2 (0–4), *p* = 0.0050] and urinary incontinence [2 (0–4) vs. 4 (2–8), *p* = 0.0047]. A shorter latency of symptomatic OH [2 (0.5–3) vs. 3 (0–5), *p* = 0.1846], urinary retention [1 (0–2.5) vs. 3 (0–5), *p* = 0.0712] and RBD [− 1 (− 3 and − 1) vs. 0 (− 2.5 and 2), *p* = 0.0852] was reported without reaching statistical significance (Table 2 and Fig. 1A).

Considering frequency of early symptoms and signs (presenting within 3 years of disease onset), patients with early stridor onset more frequently reported early urinary urgency/frequency (85.4% vs. 66.0%, *p* = 0.034), early urinary retention (48.8% vs. 38.0%, *p* = 0.039), early symptomatic OH (68.3% vs. 42.0%, *p* = 0.012) and early RBD (87.8% vs. 70.0%, *p* = 0.004) (Table 2 and Fig. 1B).

Concerning milestones of disease progression, the early stridor onset group showed a shorter latency of frequent falls [3 (2–4.5) vs. 4 (3–6), *p* = 0.0390], urinary catheterization [3 (1–4) vs. 5.5 (4–8), *p* = 0.0004], unintelligible speech [5 (4–5) vs. 7 (5–8), *p* = 0.0122], severe dysphagia/PEG [4 (4–5) vs. 8 (5–10), *p* = 0.0289] and wheelchair dependency [4 (3–5) vs. 7 (5–8), *p* = 0.0003], when compared to the late stridor onset group. However, the late stridor onset group more frequently reached unintelligible speech and severe dysphagia/PEG during the disease course (Table 2 and Fig. 1A).

In the stridor subgroup, MSA patients with early stridor onset (*n* = 15) showed higher CSF NfL levels than those with late stridor onset (*n* = 30), but without reaching statistical significance [3825 (2420–4903) vs. 3169.5 (2399–4448), *p* = 0.0980] (Table 2).

### Comparison between MSA patients with early stridor and/or RBD onset and those with late stridor and RBD onset

As previous results on this cohort showed a more rapid progression of disease in MSA patients with RBD predating disease onset [12], we repeated the analysis, in all sample, combining data on patients with early stridor onset and/or early RBD onset. Patients with early stridor and/or RBD onset (*n* = 132) were compared with those with late stridor and RBD onset (*n* = 76). Features of these two subgroups are shown in Table 3.

**Table 3:** Clinical features, latency of signs/symptoms onset and milestones of disease progression in MSA patients with early onset of stridor and/or RBD and those with late onset of stridor and RBD

	Patients with early onset (≤ 3 years) of stridor and/or RBD	Patients with late onset (> 3 years) of stridor and RBD	*p* value
	132	76	
Males,* n (%)*	68 (51.52)	52 (68.4)	**0.017**
Age at MSA onset, *y*	57.5 ± 8.3	57.9 ± 8.9	0.7914
Died,* n (%)*	89 (67.4)	68 (89.5)	** < 0.001**
Disease duration, *y*	6.7 ± 2.9	8.9 ± 4.7	** < 0.001**
Long survival ^1^, *n (%)*	1 (0.8)	10 (13.2)	** < 0.001**
MSA subtype			
MSA-P,* n (%)*	63 (47.7)	47 (61.8)	0.050
MSA-C, *n (%)*	69 (52.3)	29 (38.2)	
Symptoms at MSA onset			
Parkinsonism, *n (%)*	41 (31.1)	30 (39.5)	**0.005**
Cerebellar, *n (%)*	45 (34.1)	25 (32.9)	0. 818
Autonomic, *n (%)*	80 (60.6)	35 (46.0)	0.147
Symptoms during the disease course			
Parkinsonism, *n (%)*	115 (87.1)	67 (88.2)	0.828
Latency of parkinsonism, *y*	2 (0–4)	1 (0–4)	0.238
Cerebellar, *n (%)*	115 (87.1)	59 (77.6)	0.750
Latency of cerebellar symptoms, *y*	1 (0–3)	2 (0–5)	0.319
Pyramidal signs, *n (%)*	109 (82.6)	51 (67.1)	0.051
Latency of pyramidal signs, *y*	3 (2–5)	5 (3–7)	** < 0.001**
Urinary urgency/frequency, *n (%)*	114 (86.4)	66 (86.8)	0.410
Early urgency/frequency onset, *n (%)*	101 (76.5)	47 (61.8)	**0.024**
Latency of urinary urgency/frequency, *y*	0 (0–1)	1 (0–4)	**0.0314**
Urinary retention, *n (%)*	81 (61.4)	34 (44.7)	0.130
Early urinary retention onset, *n (%)*	58 (43.9)	13 (17.1)	**0.001**
Latency of urinary retention, *y*	2 (0–4)	4 (0–6)	**0.0281**
Urinary incontinence, *n (%)*	83 (62.8)	39 (51.3)	0.242
Early urinary incontinence, *n (%)*	41 (31.1)	7 (9.2)	**0.001**
Latency of urinary incontinence, *y*	3 (1–5)	6 (4–8)	**0.004**
Symptomatic OH, *n (%)*	91 (68.9)	47 (61.8)	0. 983
Early Symptomatic OH onset, *n (%)*	70 (53.0)	23 (30.3)	**0.001**
Latency of symptomatic OH, *y*	2 (0–3)	3.5 (1–6)	**0.0025**
Milestone of disease progression			
Frequent^2^ falls, *n (%)*	78 (59.1)	39 (51.3)	0.760
Latency of frequent falls, *y*	4 (2–5)	5 (3–7)	**0.0021**
Urinary catheterization, *n (%)*	60 (45.5)	26 (34.2)	0.320
Latency of urinary catheterization, *y*	4 (2–6)	6 (5–10)	**0.0001**
Unintelligible speech, *n (%)*	42 (31.8)	19 (25.0)	0.636
Latency of unintelligible speech, *y*	6 (4–7)	8 (7–10)	**0.0045**
Dysphagia/PEG, *n (%)*	33 (25.0)	16 (21.1)	0.838
Latency of dysphagia/PEG, *y*	6 (5–8)	8 (7–13)	**0.0041**
Wheelchair dependency, *n (%)*	78 (59.1)	40 (52.6)	0.987
Latency of wheelchair dependency, *y*	5 (4–7)	7 (6–9.5)	**0.0004**
CSF NfL^3^*, pg/ml*	3260 (2366–4569)	1993 (1694–2766)	**0.0015**
Latency of CSF NfL^3^, *n*	3.5 (2–5)	6 (5–7)	**0.006**

Data are expressed as n (%), mean ± SD, or median (interquartile range)

Statistically significant *p* values are denoted in bold (*p* value ≤ 0.05)

^1^ = disease duration ≥ 15 years

^2^ = frequent was defined at least 3 falls per year or documentation of frequent or several falls

^3^ = cerebrospinal fluid sample was collected in 87 patients

*CSF* cerebrospinal fluid, *MSA* multiple system atrophy, *MSA-C* multiple system atrophy with predominant cerebellar phenotype, *MSA-P* multiple system atrophy with predominant parkinsonism phenotype, *n* sample size, *NfL* neurofilament light chain, *OH* orthostatic hypotension, *PEG* percutaneous endoscopic gastrostomy, *RBD* REM sleep behavior disorder, *VPSG* video-polysomnography, *y* years

Concerning symptoms/sign latencies during the disease course, this subgroup, compared to patients with late onset of stridor and RBD, showed an earlier onset of pyramidal signs [3 (2–5) vs. 5 (3–7), *p* < 0.001], urinary urgency/frequency [0 (0–1) vs. 1 (0–4), *p* = 0.0314], urinary retention [2 (0–4) vs. 4 (0–6), *p* = 0.0281], urinary incontinence [3 (1–5) vs. 6 (4–8), *p* = 0.004] and symptomatic OH [2 (0–3) vs. 3.5 (1–6), *p* = 0.0025] (Table 3 and Fig. 1C).

Patients with early stridor and/or RBD onset showed less frequently parkinsonian features as mode of disease onset and more frequently reported early urinary urgency/frequency, early urinary retention, early urinary incontinence and early symptomatic OH (Table 3 and Fig. 1D).

MSA patients with early stridor and/or RBD onset demonstrated a more rapid progression with shorter latency of all milestones of disease progression: frequent falls [4 (2–5) vs. 5 (3–7), *p* = 0.0021], urinary catheterization [4 (2–6) vs. 6 (5–10), *p* = 0.0001], unintelligible speech [6 (4–7)vs. 8 (7–10), *p* = 0.0045], dysphagia/PEG [6 (5–8) vs. 8 (7–13), *p* = 0.0041], wheelchair dependency [5 (4–7) vs. 7 (6–9.5), *p* = 0.0004] (Table 3 and Fig. 1C).

On the whole sample, the subgroup of patients with early stridor and/or RBD onset (*n* = 68), showed higher CSF NfL than those with late stridor and RBD onset (*n* = 19) [3260 (2366–4569) vs. 1993 (1694–2766), *p* = 0.0015], despite latency from disease onset to CSF collection was shorter in the first subgroup than in the second one [3.5 (2–5) vs. 6 (5–7) years, *p* = 0.006] (Table 3).

### Survival analysis

Kaplan–Meier estimates of death in the overall population are shown in Supplementary Fig. 1A. In this analysis, the median duration of illness was 7.47 years.

The risk of death estimated by Kaplan–Meier analysis (Supplementary Fig. 1B) was higher in patients with early stridor than those with late stridor (*p* < 0.0001, log-rank test), with an incidence rate of death of 13.5 per 100 person-years in the first group and 9.7 per 100 person-years in the second group.

MSA patients with early RBD onset showed a higher risk of death than patients with late RBD onset (*p* < 0.0001, log-rank test) (Supplementary Fig. 1C), with an incidence rate of death of 9.8 per 100 person-years in the first group and 6.6 per 100 person-years in the second group.

Considering latencies of both stridor and RBD, the Kaplan–Meier analysis (Supplementary Fig. 1D) showed a higher risk of death in patients with early stridor and/or RBD onset than those with late stridor and RBD onset (*p* = 0.048, log-rank test).

The univariate and multivariate Cox regression analyses identified the following as factors associated with short survival: age at disease onset [HR = 1.05 (1.03–1.07), *p* < 0.001], autonomic disease onset [HR = 1.54 (1.10–2.14), *p* = 0.011], early symptomatic OH [HR = 1.96 (1.41–2.72), *p* < 0.001], early urinary urgency/frequency [HR = 1.66 (1.17–2.36), *p* = 0.004], early urinary retention [HR = 2.47 (1.52–4.02), *p* < 0.001], early incontinence [HR = 2.39 (1.48–3.85), *p* < 0.001], early stridor [HR = 2.16 (1.44–3.25), *p* < 0.001] and early RBD [HR = 2.98 (1.62–5.46), *p* < 0.001] (Table 4). In the multivariable model, early stridor onset [HR = 1.72 (1.04–2.86), *p* = 0.036] and early RBD onset [HR = 2.67 (1.35 – 5.27), *p* = 0.005] remained independent predictor of mortality after adjustment for autonomic onset and age at disease onset.

**Table 4:** Variables associated with survival in MSA patients in the univariate Cox regression analysis

Variable	*n*	Unadjusted H-Ratio (95% CI)	*p* value
Age at disease onset	208	1.05 (1.03–1.07)	** < 0.001**
Sex			
Female	88	0.83 (0.60–1.16)	0.274
Male	120	1.0 (reference)	
Clinical phenotype			
MSA-P	110	0.99 (0.72–1.36)	0.954
MSA-C	98	1.0 (reference)	
Symptom of disease onset			
Autonomic	115	1.54 (1.10–2.14)	**0.011**
Cerebellar	70	0.89 (0.63–1.25)	0.501
Parkinsonism	71	1.07 (0.77–1.50)	0.681
Symptom of disease onset			
Early symptomatic OH	93	1.96 (1.41–2.72)	** < 0.001**
Early urinary urgency/frequency	148	1.66 (1.17–2.36)	**0.004**
Early urinary retention	71	2.47 (1.52–4.02)	** < 0.001**
Early urinary incontinence	48	2.39 (1.48–3.85)	** < 0.001**
Stridor (VPSG)			
Yes	91	0.99 (0.71–1.39)	0.979
No	117	1.0 (reference)	
Early stridor (VPSG)			
Yes	41	2.16 (1.44–3.25)	** < 0.001**
No	50	1.0 (reference)	
Early RBD (VPSG)			
Yes	127	2.98 (1.62–5.46)	** < 0.001**
No	33	1.0 (reference)	
NfL	87	1.00 (0.99–1.00)	0.644

Statistically significant *p* values are denoted in bold (*p* value ≤ 0.05)

*CI* confidence interval, *H-Ratio* hazard ratio, *MSA-C* multiple system atrophy with predominant cerebellar phenotype, *MSA-P* multiple system atrophy with predominant parkinsonism phenotype, *n* sample size, *NfL* neurofilament light chain, *OH* orthostatic hypotension, *RBD* REM sleep behavior disorder, *VPSG* video-polysomnography

## Discussion

Our study assessed, for the first time, that patients with early stridor onset alone and with early sleep disorders (combining early stridor onset and/or early RBD onset) showed not only a shorter survival but also a more severe and more rapid disease progression, determining the impact of this domain on MSA severity, progression and prognosis. Further, we demonstrated that these more severe phenotypes of MSA showed higher CSF NfL levels.

In the stridor subgroup, patients developing stridor within the first 3 years of disease, more frequently presented with autonomic onset and reported early urinary urgency/frequency, early urinary retention, early symptomatic OH and early RBD. Moreover, this subgroup developed a more progressive and severe disease, showing a shorter latency of all milestones of disease progression.

Patients with early stridor and/or RBD onset showed a more rapid and severe disease progression characterized by a rapid involvement of autonomic features (both symptomatic OH and urinary dysfunctions) and an early achievement of milestones of disease progression. In particular, MSA patients with early stridor and/or RBD onset, compared to patients with late onset of stridor and RBD, showed an earlier onset of pyramidal signs, urinary urgency/frequency, urinary incontinence and symptomatic OH, reaching all 5 milestones of disease progression with shorter latency (frequent falls, urinary catheterization, unintelligible speech, dysphagia/PEG and wheelchair dependency).

Although autonomic failure is a negative predictor on prognosis per se, we demonstrated that both early stridor and early RBD are independent risk factors on MSA survival.

Another result of our study is that these more severe phenotypes of MSA showed higher NfL levels: 1- on the whole sample, MSA patients with early stridor and/or RBD onset, showed higher CSF NfL than those with late stridor and RBD onset; 2- in the stridor subgroup, patients with early stridor onset showed higher CSF NfL levels than those with late stridor onset even if without reaching statistical significance. These findings reinforce the concept of a more aggressive disease trajectory in these subgroups and underscore the pivotal role of early sleep disorder manifestations in MSA progression. Consistent with previous literature, baseline NfL levels have been shown to correlate with disease severity and serve as predictors of rapid progression, reduced survival, and the extent of neurodegeneration in MSA [13, 22, 23], as well as in other neurodegenerative disorders such as amyotrophic lateral sclerosis [24, 25].

While NfL in MSA remained stable over time with serial CSF measurements [23, 26], their levels correlate with disease severity particularly in the earlier stages of disease [13, 27]. Higher NfL levels, reliable biomarkers of axonal damage across a variety of neurological disorders, reflect a more disease burden and neuronal pool impairment [24] and could be a potential biomarker for patients’ stratification in MSA drug trials [13].

Overall, our clinical and instrumentals findings impact the clinical approach, improving patient care, but also open up new scientific perspectives.

As per the clinical approach, MSA patients presented with early sleep disorders require a careful assessment and a closer follow-up of autonomic dysfunction, laryngeal muscles and vocal cords abnormalities, respiratory disorders and clinical features leading to milestones of disease progression (e.g., dysphagia, urinary retention, symptomatic OH, frequent falls) and to higher risk of death (e.g., urinary and respiratory infections, cardiovascular failure).

From a research point of view, these results generate different implications on patient’s selection and outcome interpretation in trial focusing on MSA. Efforts toward disease-modifying therapies in MSA have seen a remarkable surge in recent years. The heterogeneity of MSA presentation and progression, along with the relatively short disease course from symptom onset to death, complicates clinical outcome measures and the construction of study design. Improving the understanding of disease progression and factors affecting the disease course in MSA could be useful, in view of future disease-modifying therapies, to select more homogeneous subgroups of patients for clinical trials.

Despite studies on trajectories of disease and on predictors of survival did not include sleep disorders as clinical variable [2–4, 8], our study suggests that sleep disorders are key features of MSA, playing a role in presentation, prognosis and progression of disease.

The evidence of a more rapid and severe disease progression in MSA patients who early developed stridor, RBD and autonomic involvement could define a different MSA phenotype with a widespread impairment of central-brainstem circuits and a greater axonal degeneration, as suggested by higher CSF NfL levels.

The bidirectional relationship between RBD and stridor onset (an earlier onset of stridor was found in patients with early RBD and vice versa) and their mutual association with early autonomic onset could also shed light on a common pathogenic pathway residing in key brainstem areas involved in sleep, cardiovascular control and automatic respiration regulation [28]. This correlation could be linked to the highly topographic and functional interconnection of brainstem neuronal networks (parabrachial nucleus, pre-Botzinger complex, rostral ventrolateral medulla, pontine micturition center, pedunculopontine tegmental nucleus, sublaterodorsal tegmental nucleus, and locus ceruleus) whose degeneration in MSA has been widely documented [28–30]. Therefore, the early occurrence of RBD and stridor during the disease course entails an early involvement of brainstem nuclei, which leads to early autonomic dysfunction and other sleep and breathing disorders.

The strengths of our study are that all patients were seen and diagnosed in a single tertiary center with long-standing expertise in autonomic, sleep and movements disorders, ensuring uniformity of data. Patients recruited in the prospective cohort were evaluated every 6 months, those included in the retrospective cohort were evaluated at least once a year during the disease course and, in both cohorts, data were systematically collected. Moreover, cardiovascular autonomic failure, stridor and RBD were instrumentally documented.

The present study has some limitations. Data on quality of life and Unified Multiple System Atrophy Rating Scale (UMSARS) were not systematically collected at baseline and during subsequent follow-up visits. NfL evaluation was not performed in all sample but in 87/208 MSA patients (45/91 MSA in the stridor subgroup). Moreover, CSF collection for NfL analysis was performed, for each patient, during the first inpatient evaluation, with a median latency from disease onset of 4 years (IQR = 3–6, range = 1–10 years). However, previous studies, also of our group, demonstrated that the NfL levels remained stably elevated during disease course and were not associated with disease duration [13, 23, 26]. Data on causes of death were collected by talking with relatives when medical records were not available and were well-defined in 95 of the 157 deceased patients (60.5%). No differences on causes of death were found between subgroups but, for the non-negligible percentage of missing data (39.5%), this analysis should be taken with caution. Finally, the low number of neuropathologically established MSA may be considered the most significant limitation of our study. However, 174 out 208 patients were classified as clinically established MSA and the specificity of this category according to neuropathological studies is 99–100% [31, 32].

Current trials on MSA have set inclusion criteria that not only aim to ensure a correct diagnosis, but also to recruit patients with limited functional impairment in order to capture a still responsive disease stage and to avoid a ceiling effect of clinical outcome measures [33–35]. These factors underline the need for enhancing patients’ stratification and disease trajectories at an early stage. We showed, with a systematic clinical and instrumental assessment, in a large sample of MSA patients, that early onset of sleep disorders (sleep-related stridor alone or in association with RBD) underlines a widespread impairment of central-brainstem circuits with increase axonal degeneration, leading a greater impairment of the central autonomic networks, an early achievement of milestones of disease progression and disability, and a short survival. Our findings could contribute to define a more severe MSA phenotype, helping to better recognize patients with a poor prognosis and to stratifying them for domain of disease onset, especially in view of upcoming trials.

## Supplementary Information

Below is the link to the electronic supplementary material.

Supplementary file1 (TIF 393 KB)

## Data Availability

Anonymized data not published within this article will be made available by request from any qualified investigator.
