# Supplementary figures and images for: Early onset sleep disorders predict severity, progression and death in multiple system atrophy
Source: J Neurol. 2025 Mar 1;272(3):239. doi: 10.1007/s00415-025-12969-6 (PMC11872749; doi:10.1007/s00415-025-12969-6)

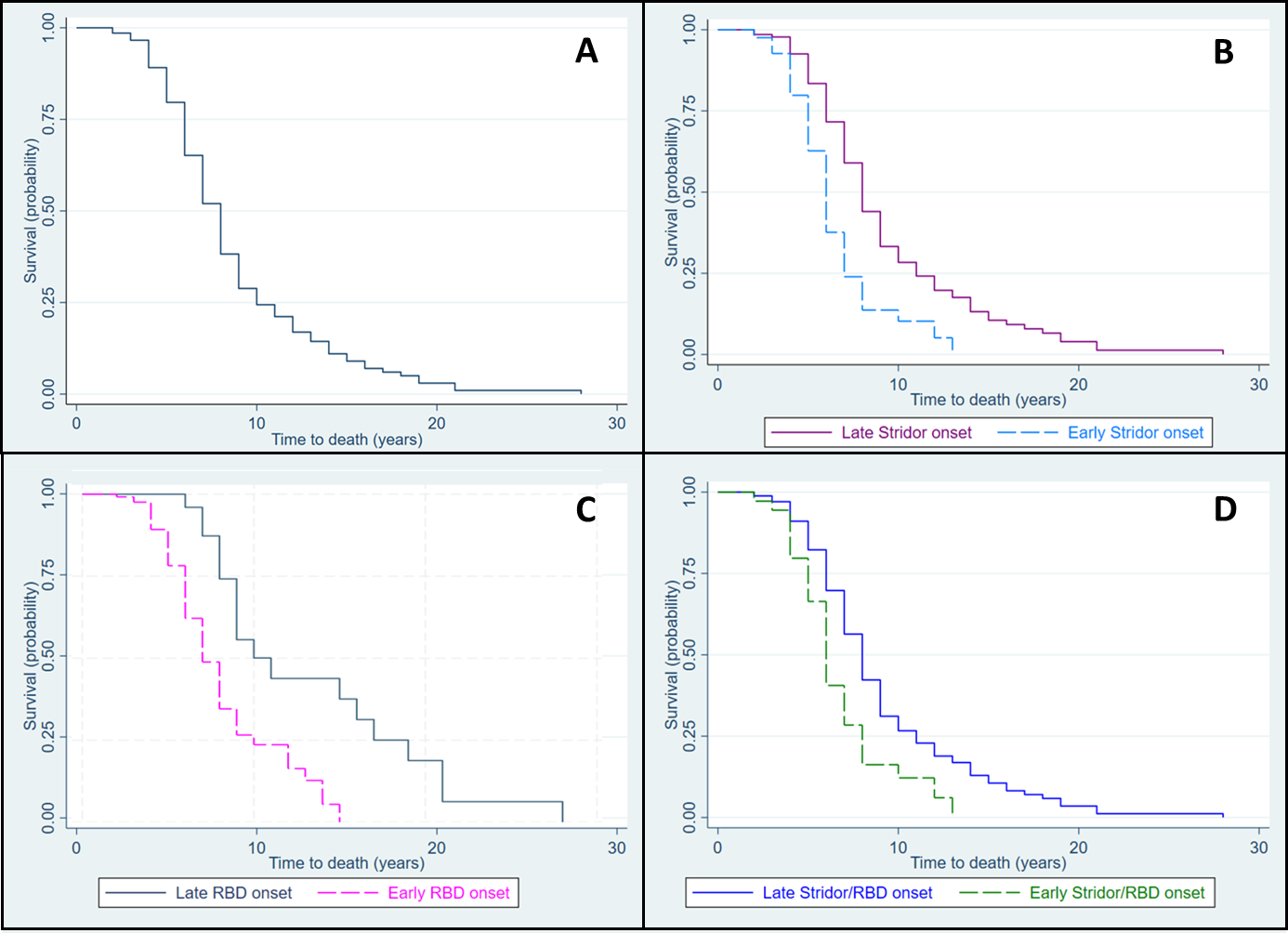

Supplement: Supplementary file 1 — Supplementary file1 (TIF 393 KB) [file 415_2025_12969_MOESM1_ESM.tif]
